# Development and interpretation of a machine learning predictive model for early cognitive impairment in hypertension associated with environmental factors

**DOI:** 10.3389/fcvm.2025.1477185

**Published:** 2025-08-29

**Authors:** Xia Zhong, Tianen Zhao, Shimeng Lv, Guangheng Zhang, Jing Li, Donghai Liu, Huachen Jiao

**Affiliations:** ^1^ Institute of Child and Adolescent Health, School of Public Health, Peking University, Beijing, China; ^2^ Purchasing Department, Jinan Lixia Dezhengtang Hospital of Traditional Chinese Medicine, Jinan, Shandong, China; ^3^ Institute of First Clinical Medical College, Shandong University of Traditional Chinese Medicine, Jinan, Shandong, China; ^4^ College of Laboratory Animal Science, Shandong First Medical University, Jinan, Shandong, China; ^5^ Institute of Cardiology Department, Affiliated Hospital of Shandong University of Traditional Chinese Medicine, Jinan, Shandong, China

**Keywords:** environmental exposure factor, cognitive impairment, hypertension, predictive model, machine learning

## Abstract

**Background and objective:**

Risk-based predictive models are a reliable tool for early identification of hypertensive cognitive impairment. However, the evidence of the combination of individual factors and natural environmental factors is still insufficient. The aim of this study was to establish a well-performing machine learning (ML) model based on personal and natural environmental factors to help assess the risk of early cognitive impairment in hypertension.

**Methods:**

In this study, a total of 757 Chinese hypertensive patients from from different regions of Shandong Province, China (aged 31–95, male 49.01%) were randomly divided into training group (70%) and verification group (30%). Modelling variables were determined by a 5-fold cross-validated least absolute shrinkage and selection operator (LASSO) regression analysis. Five ML classifiers, XGB (extreme gradient boosting), LR (logistic regression), AdaBoost (adaptive boosting), GNB (gaussian naive bayes), and SVM (support vector machines), have been developed. Area under the ROC curve (AUC), accuracy, sensitivity, specificity, and F1 scores were used to access the model performance. Shape Additive explanation (SHAP) models reveal the feature importance. The clinical performance of the model was evaluated by Decision Curve Analysis (DCA).

**Results:**

Cognitive impairment was diagnosed in 17.44% (*n* = 132). LASSO regression analyses suggested that age, waist circumference, urban green coverage, educational levels, annual sunshine hours, and area whole-day average noise were considered significant predictors of early cognitive impairment in hypertension. The obtained XGBoost model yielded good predictive performance with the AUC (0.893), F1 score (0.627), accuracy (0.837), sensitivity (0.780), and specificity (0.853). The predictive model's clinical net benefit was confirmed through DCA analysis.

**Conclusion:**

The XGBoost model developed based on personal factors and natural environmental factors can predict early cognitive impairment of hypertension with superior predictive performance. Larger population cohorts are needed in the future to validate these findings and potentially enhance the ability to identify the occurrence of early cognitive impairment in people with hypertension.

## 1 Introduction

Dementia, an increasingly prevalent and challenging neurodegenerative disease, affects about 50 million people worldwide (1), a number that is expected to triple by 2050 (2). Cognitive impairment is the preclinical stage of dementia, and active prevention can reduce the likelihood of developing dementia. Hypertension is one of the major risk factors for cognitive impairment (3) and is associated with a 1.62-fold increase in the risk of cognitive impairment (4). Currently, there is no conclusive evidence supporting the role of pharmacological therapies in preventing cognitive decline (5). In recent years, there has been a rapid increase in the number of patients with hypertensive cognitive disorders seeking medical help and counselling. The specific mechanisms that trigger and promote cognitive decline are unclear, and treatment options are limited, so there is an urgent need to develop more precise treatment strategies for hypertensive cognitive disorders.

Risk factors for cognitive impairment have not been fully explored. Most studies have focused on individual factors such as aging, smoking, physical inactivity, low levels of education, obesity, vitamin D deficiency, diabetes, and hypertension (6, 7), but these cannot fully explain cognitive impairment. Increasing evidence supports the need to focus the risk of cognitive impairment on environmental factors. In addition, it is increasingly recognized that environmental factors may hold promise for future predictive methods and are more universal than brain imaging markers and cerebrospinal fluid (CSF). Several studies have reported the relationship between natural environmental factors and cognitive impairment, including climate (8), greenness (9), air pollution (10), noise exposure (11), etc. These modifiable natural environmental factors are considered promising predictors of cognitive impairment, despite varying reported results.

Recently, related risk factors and predictive models for hypertensive cognitive impairment developed based on these risk factors have been preliminarily explored, especially in the Chinese population. Zhang et al. (12) showed that plateau environment, age, abdominal circumference, and serum uric acid (SUA) were independent risk factors for hypertensive cognitive impairment. Li et al. (13) reported that duration of hypertension, systolic blood pressure (SBP), homocysteine (Hcy), and SUA were risk factors for cognitive dysfunction, and duration of education was negatively correlated with cognitive dysfunction. Ma et al. (14) also revealed that low levels of education, elevated body mass index (BMI) and waist- to- height ratio (WHR) were independent risk factors for hypertensive cognitive impairment. Lu et al. (15) developed a predictive model of hypertensive cognitive impairment based on a number of influencing factors including hypertension grade, smoking, sleep disorder, and duration of hypertension, and the AUC, sensitivity, and specificity of the model were 0.765, 0.630, and 0.877, respectively. Currently recognized cognitive impairment is more likely to involve a combination of factors (5), and prevention strategies based on modifiable factors appear to be more important. To the best of our knowledge, a predictive model based on personal and natural environmental factors for early cognitive impairment in hypertensive patients has not been developed.

Machine learning (ML) can be based on important information modeling, which helps reveal the relationship between factors and diseases in complex data environments (16). ML techniques have shown benefits in developing risk prediction models for cardiovascular and cerebrovascular events (17–20). Furthermore, predictive models for cognitive impairment based on ML algorithms have also been reported (21–24). However, evidence for ML-based risk prediction models for early cognitive impairment in hypertension remains limited, especially when environmental factors are involved.

Therefore, we aimed to develop for the first time a superior ML prediction model that considers modifiable personal and environmental exposure factors to predict the risk of early cognitive impairment in hypertension, with the hope of providing optimal strategies for the early diagnosis and management of hypertensive cognitive impairment.

## 2 Methods

### 2.1 Participants and study design

**Figure 1:**
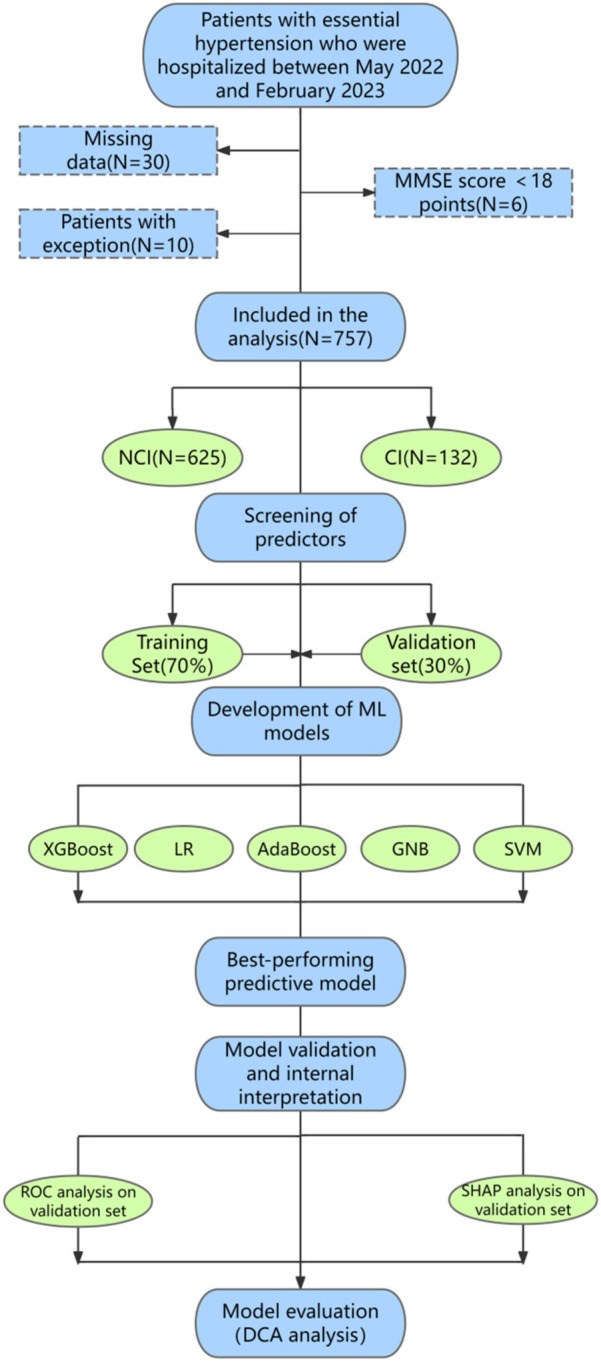
Basic research flow diagram. Flowchart shows the details of participant selection and ML model development and validation. A total of 757 participants were included according to standard procedures, including 625 individuals with NCI and 132 individuals with early cognitive impairment. 757 participants were randomly divided into two groups: 70% for training and 30% for validation. In this study, the model was trained and validated for 10 repetitions using a five-fold CV. Finally, we developed predictive models using five classifiers, including XGBoost, Logistic, AdaBoost, GNB, and SVM, and further explored the performance of the predictive model through ROC curve, SHAP model, and DCA analysis. MMSE, mini-mental state examination; NCI, no cognitive impairment; CI, cognitive impairment; LR, logistic regression; GNB, Gaussian naive Bayes; SVM, support vector machines; ML, machine learning; ROC, receiver operating characteristic; SHAP, shape additive explanation; DCA, decision curve analysis.

This study received approval from the Institutional Review Board (IRB) of the Affiliated Hospital of Shandong University of Traditional Chinese Medicine [Approval Number: (2023) Ethics Review No. (109)-KY] with the informed consent of all participants. In this study, the research area is Shandong Province, China (115–120°E and 35–38°N). We collected 803 hypertension patients from 8 hospitals in 5 prefecture-level cities by stratified sampling between May 2022 and February 2024. Finally, a total of 757 hypertensive patients recruited from four prefecture-level hospitals in Yantai, Jinan, Weifang and Dongying in Shandong Province were eligible for the development and validation of the prediction model. Participants were included if they were (1) 30 years of age or older; and (2) essential hypertension. Participants were excluded if they (1) had a history of neurological disease (e.g., Parkinson's disease, stroke, epilepsy, brain tumor, brain trauma, mental or psychiatric illness, and dementia) or (2) had a history of cardiovascular disease (e.g., severe arrhythmia, heart failure, and cardiac surgery) or (3) had severe vision or hearing impairment. A detailed study flow diagram of the selection of participants is shown in Figure 1. For better analysis, we excluded 30 individuals with missing data, 10 individuals with abnormal data, and 6 individuals with the Mini-Mental State Examination (MMSE) scores below 18 points. Data collection and quality control were standardized across all centers through electronic medical records and centralized the Standard Operating Procedure (SOP) training, with key variables (e.g., MMSE) demonstrating high inter-site reliability (Kappa >0.8, ICC >0.9).

### 2.2 Assessment of hypertension and early cognitive impairment

All patients were diagnosed by experienced cardiologists using the following criteria. Diagnostic criteria for hypertension are (25): (1) systolic blood pressure (SBP) ≥ 140 mmHg, or (2) diastolic blood pressure (DBP) ≥ 90 mmHg, (3) and/or the use of antihypertensive medications. In addition, early cognitive impairment was determined by the Chinese version of the MMSE assessment questionnaire. MMSE scores range from 0 to 30, with low scores representing poor cognitive function (26). MMSE scores above 27 are considered normal cognitive function, and MMSE scores above 18 and below 27 are identified as early cognitive impairment (27, 28).

### 2.3 Predictors and feature selection

Based on previously reported cognitive impairment factors, personal factors (age, sex, estimated duration of hypertension, levels of education, type of work, smoking, drinking, BMI, waist circumference, waist-to-hip ratio) and natural environmental factors (air temperature, annual sunshine hours, urban precipitation, relative humidity, area whole-day average noise value, traffic average noise value, per capita public green areas, urban green coverage) were analyzed. Basic information about the participants, including age, sex, estimated duration of hypertension, education levels, type of work, smoking and drinking, was obtained through questionnaires. Anthropometric indicators including weight (kg), height (cm), hip circumference (cm) and waist circumference (cm) were standardized measurements. BMI was obtained by dividing weight into kilograms by height in meters squared in kg/m^2^ (29). WHR was obtained by dividing waist circumference (cm) by hip circumference (cm). We obtained annual average data from the Shandong Meteorological Bureau and local authoritative environmental monitoring stations for 2018–2022, including air temperature, annual sunshine hours, urban precipitation, relative humidity, area whole-day average noise value, traffic average noise value, per capita public green areas, urban green coverage. Air temperature (℃) is the temperature of the air and is calculated by dividing the average monthly temperature over 12 months by 12. Annual sunshine hours (h) are the number of hours the sun actually hits the ground and are calculated by adding up the number of hours of sunshine over 12 months. Precipitation (mm) is the depth of liquid or solid water (after melting) that falls from the sky to the ground and accumulates on the ground without evaporation, penetration, or loss and is calculated by the cumulative amount of monthly precipitation over 12 months. Relative humidity (%) is the ratio of the actual water pressure in the air to saturated water pressure at the prevailing temperature and is calculated by adding up the average monthly humidity over 12 months and dividing by 12. Monitoring sites used to measure noise include major urban traffic intersections and residential areas. Area whole-day average noise value (db) mainly includes industrial noise, traffic noise, construction noise, social noise. Traffic average noise value (db) depends mainly on the load or traffic flow on the road (30). Traffic flow is determined by the number of vehicles passing through the center of the intersection in a unit of time. Per capita public green areas (m^2^) in built-up areas refers to the average area of public green space occupied by each resident of a city. Urban green coverage rate (%) is obtained by the ratio of green coverage area to built-up area in urban built-up area.

We chose LASSO regression for feature selection because of its ability to handle high-dimensional, small sample datasets through L1 regularization, which automatically eliminates irrelevant features by shrinking their coefficients to zero while retaining critical predictors (31). This approach balances sparsity and computational efficiency, addressing overfitting risks inherent in limited data. Though recursive feature elimination (RFE) and Shape Additive explanation (SHAP) -based methods offer interpretability (32, 33), their limitations outweighed their benefits: Computational Costs: RFE's iterative process and SHAP's prediction dependency become infeasible with large feature sets. Noise Sensitivity: Both methods struggle with redundant/noisy features common in small samples, risking false-positive selections. Scalability Issues: RFE's recursive elimination and SHAP's permutation evaluations degrade performance as feature counts increase. LASSO's integrated feature selection and regularization mechanism eliminated iterative processing needs, making it uniquely suited for our dataset.

### 2.4 Outcomes

In this study, a total of 757 patients with hypertension were included for analysis, of which 132 (17.44%) participants were identified as having early cognitive impairment. Finally, we screened 6 core predictors from 18 variables, including 3 personal parameters (age, waist circumference, educational levels) and 3 natural environmental parameters (urban green coverage, annual sunshine hours, and area whole-day average noise value), to develop and validate the predictive model of cognitive impairment in hypertension.

### 2.5 Development and validation of Ml models

The flow of machine learning model development and validation is shown in Figure 1. First, we selected predictors and randomly assigned participants to two groups: 70% for model development and training, and 30% for model evaluation and validation to prevent model overfitting. A five-fold CV was used to train and verify the model. Then, ROC curve analysis was performed to evaluate and compare the predictive performance of five ML classifiers [XGBoost, logistic regression (LR), AdaBoost, GNB, and SVM], including AUC, sensitivity, specificity, accuracy, and F1 scores. Finally, we further explored the significance of predictors and the clinical applicability of the predictive model through SHAP and DCA analysis. According to the classification confusion matrix (34), the ML model is defined as true positive (TP) and true negative (TN) if it can correctly predict cognitive impairment; conversely, the ML model was defined as false positive (FP) or false negative (FN). Sensitivity is defined as the percentage of samples that test positive and is calculated by the formula: Sensitivity = TP/(TP + FN). Specificity is defined as the percentage of samples that are actually negative that are judged to be negative, and the formula is calculated as follows: specificity = TN/(TN + FP). Accuracy refers to the proportion of samples correctly classified for a given data in the total samples, and its calculation formula is: Accuracy = (TP + TN)/(TP + TN + FP + FN). F1 score is used to measure the overall performance of the classifier, and its formula is as follows: F1 score = 2×precision × recall/(precision + recall).

### 2.6 Statistical analyses

Continuous data were represented by mean ± standard deviation (SD) or median [25th, 75th], while categorical data were expressed by number (%). We performed the t-test, Mannwhitney-U test, Analysis of Variance (ANOVA), and Chi-square test to select variables with comparative differences between groups for further LASSO regression dimension reduction, and screened predictors for modeling through five-fold cross-validation (CV). All patients were randomly assigned to a training or validation group (7:3), and predictive models were developed using five classifiers (XGBoost, LR, AdaBoost, GNB, and SVM). We developed ROC curves and compared the AUC, accuracy, sensitivity, specificity, and F1 scores of the five models. Finally, we used SHAP analysis to determine the importance of features in the predictive model, and developed DCA curves to evaluate the clinical applicability of the predictive model. All statistical analysis results were obtained using Python version 3.7 and R 3.6.3. A *p* < 0.05 was considered statistically significant, and a 2-sided test was performed.

## 3 Results

### 3.1 Comparison of personal and environmental factors between early cognitive impairment and controls

Table 1 shows the personal and environmental factors for all patients. The mean age of all participants was 67.11 ± 11.47 years, with 48.98% male. Of the 757 participants, 132 (17.44%) had early cognitive impairment. Compared to NCI, individuals with early cognitive impairment were older, had a longer estimated duration of hypertension, lower educational attainment, and larger waist circumference, and were more likely to perform manual labor (all *p* < 0.05). In addition, participants with early cognitive impairment lived in areas with lower temperatures and urban green coverage, fewer hours of annual sunshine, higher relative humidity, and area whole-day average noise value (all *p* < 0.05). However, there were no significant differences between the two groups in gender, smoking, drinking, BMI, waist-to-hip ratio, urban precipitation, traffic average noise value, and per capita public green areas (all *p* > 0.05).

**Table 1:** Comparison of personal and environmental factors between cognitive impairment and controls.

Indicators	Total (*N* = 757)	NCI (*N* = 625)	CI (*N* = 132)	*P* value
Personal factors				
Age, years	67.11 ± 11.47	65.27 ± 11.21	75.83 ± 8.22	<0.001*
Sex (men), *n* (%)	371 (49.01)	307 (49.12)	64 (48.49)	0.894
Estimated duration of hypertension, months, median [IQR]	117.00 [59.00, 179.00]	109.00 [58.00, 176.00]	134.00 [67.00, 212.00]	0.017*
Educational levels, *n* (%)				<0.001*
Primary school or below	299 (39.50)	209 (33.44)	90 (68.18)	
Junior high school or senior high school	411 (54.29)	369 (59.04)	42 (31.82)	
University or above	47 (6.21)	47 (7.52)	0 (0.00)	
Type of work, *n* (%)				<0.001*
Manual	406 (53.63)	306 (48.96)	100 (75.76)	
Mental	104 (13.74)	100 (16.00)	4 (3.03)	
Both manual and mental	247 (32.63)	219 (35.04)	28 (21.21)	
Current drinker, *n* (%)	195 (25.76)	168 (26.88)	27 (20.46)	0.125
Current smoker, *n* (%)	231 (30.52)	199 (31.84)	32 (24.24)	0.085
BMI, kg/m^2^, mean ± SD	24.97 ± 3.30	24.87 ± 3.31	25.45 ± 3.19	0.068
Waist circumference, cm, mean ± SD	84.02 ± 15.38	83.28 ± 15.76	87.53 ± 12.83	0.001*
Waist-to-hip ratio, mean ± SD	0.86 ± 0.12	0.86 ± 0.13	0.87 ± 0.09	0.494
Environmental factors				
Air temperature, ℃, mean ± SD	13.92 ± 0.83	13.96 ± 0.85	13.71 ± 0.74	<0.001*
Annual sunshine hours, h, mean ± SD	2,374.66 ± 39.90	2,376.05 ± 40.84	2,368.08 ± 34.35	0.021*
Urban precipitation, mm, mean ± SD	775.17 ± 23.04	775.35 ± 23.95	774.28 ± 18.09	0.562
Relative humidity, %, mean ± SD	64.10 ± 3.17	63.97 ± 3.22	64.73 ± 2.85	0.007*
Area whole-day average noise value, db, mean ± SD	54.46 ± 0.93	54.42 ± 0.97	54.65 ± 0.69	0.001*
Traffic average noise value, db, mean ± SD	67.38 ± 0.71	67.40 ± 0.72	67.28 ± 0.61	0.051
Per capita public green areas, sq.m/person, mean ± SD	16.34 ± 1.64	16.30 ± 1.69	16.53 ± 1.37	0.092
Urban green coverage, %, mean ± SD	41.02 ± 0.43	41.03 ± 0.44	40.92 ± 0.32	<0.001*

NCI, no cognitive impairment; CI, cognitive impairment; BMI, body mass index.

*Statistically significant (*P* < 0.05).

### 3.2 Predictors were screened by LASSO regression analysis

**Figure 2:**
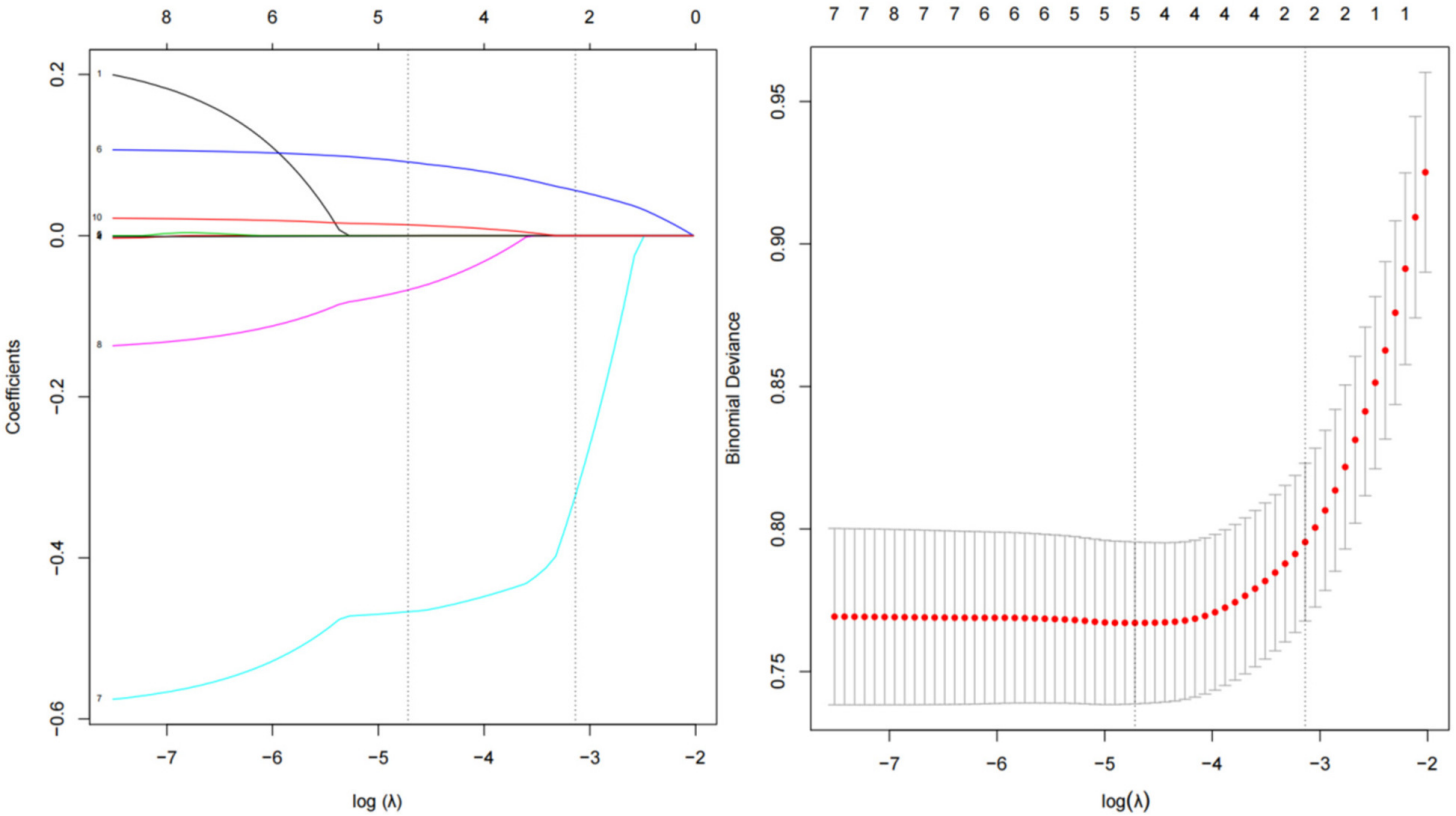
Coefficient plot and adjustment parameters in LASSO models. Each line in the figure represents the trajectory of a standard coefficient of an influence factor, and the number at the top of the figure is the number of remaining non-zero coefficient variables. The red dot and the upper and lower ends of the line segment represent the mean and range of the binomial deviation corresponding to each log (*λ*) value, and the black dashed line corresponds to the log (*λ*) value as the determined optimal penalty coefficient.

For the simplicity of the model, we performed LASSO regression analysis to reduce the dimension of 10 indicators with statistical differences in Table 1, including age, estimated duration of hypertension, educational levels, type of work, waist circumference, air temperature, annual sunshine hours, relative humidity, area whole-day average noise value, and urban green coverage. As shown in Figure 2, as the log (*λ*) value increases, the normalization coefficients of the 10 candidate parameters are compressed to varying degrees until all changes are zero (35). Finally, we selected four predictors for model development, including age, education levels, waist circumference, annual sunshine hours, area whole-day average noise, and urban green coverage.

### 3.3 Development of predictive models based on five classifiers

**Figure 3:**
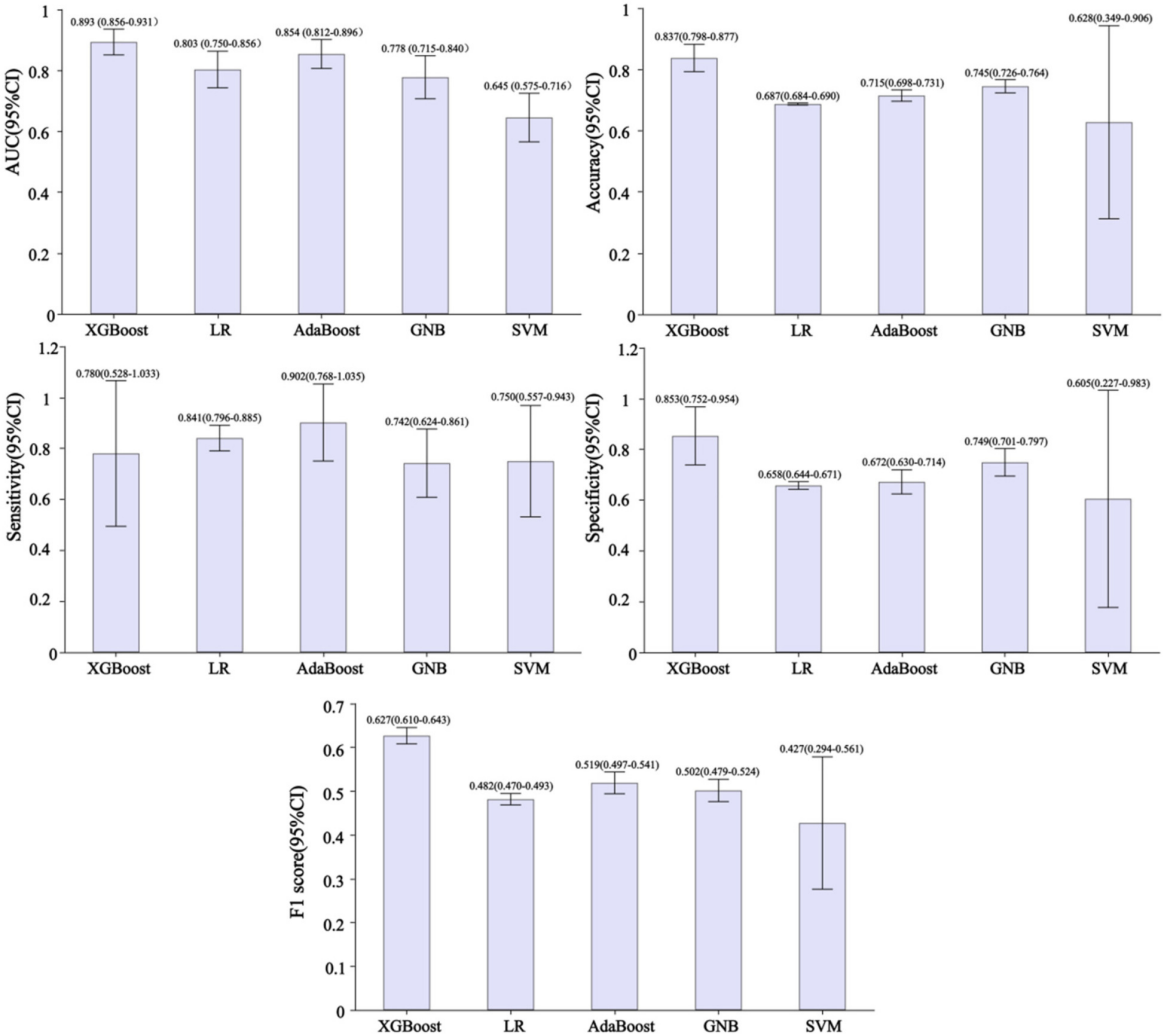
Performance of developed predictive models based on different classifiers. AUC, area under the curve; XGBoost, extreme gradient boosting; LR, logistic regression; AdaBoost, adaptive boosting; GNB, Gaussian naive Bayes; SVM, support vector machines.

**Figure 4:**
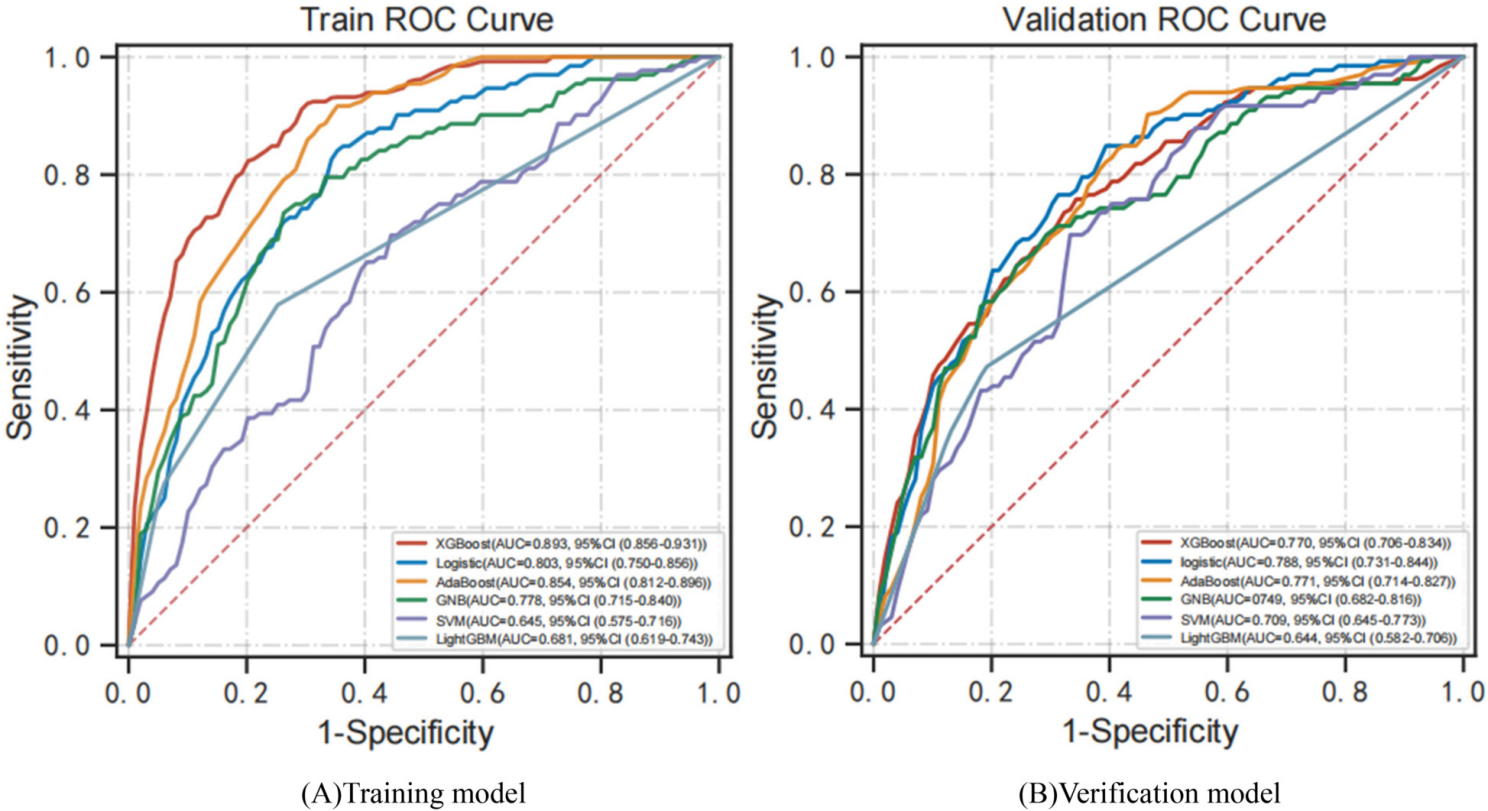
ROC curves for models developed with six predictors. **(A)** Training ROC analysis results of five classifiers. **(B)** Verification ROC analysis results for five classifiers. ROC curve, receiver operating characteristic curve; AUC, area under the curve; XGBoost, extreme gradient boosting; AdaBoost, adaptive boosting; GNB, Gaussian naive Bayes; SVM, support vector machines.

Figure 3 shows the performance of predictive models based on different classifiers. Current results suggest that the best performance model was the XGBoost model, with an AUC of 0.893, accuracy of 0.837, sensitivity of 0.780, specificity of 0.853, and F1 score of 0.627. The AdaBoost model ranked second in performance, with AUC of 0.854, accuracy of 0.715, sensitivity of 0.902, specificity of 0.672, and F1 score of 0.519. Compared with the other four models, the SVM model has poor performance, with AUC of 0.645, accuracy of 0.628, sensitivity of 0.750, specificity of 0.605, and F1 score of 0.427. Figure 4 shows the training and validation ROC curves for the five classifiers. The AUC of the XGBoost model in the training set was 0.893 (Figure 4A), and the AUC of the validation set was 0.770 (Figure 4B). The predictive performance (including accuracy, sensitivity, specificity, and F1 score) of the XGBoost model was significantly better than that of the other four classifiers (all *P*-values < 0.05).

### 3.4 Outcomes of XGBoost model evaluation

**Figure 5:**
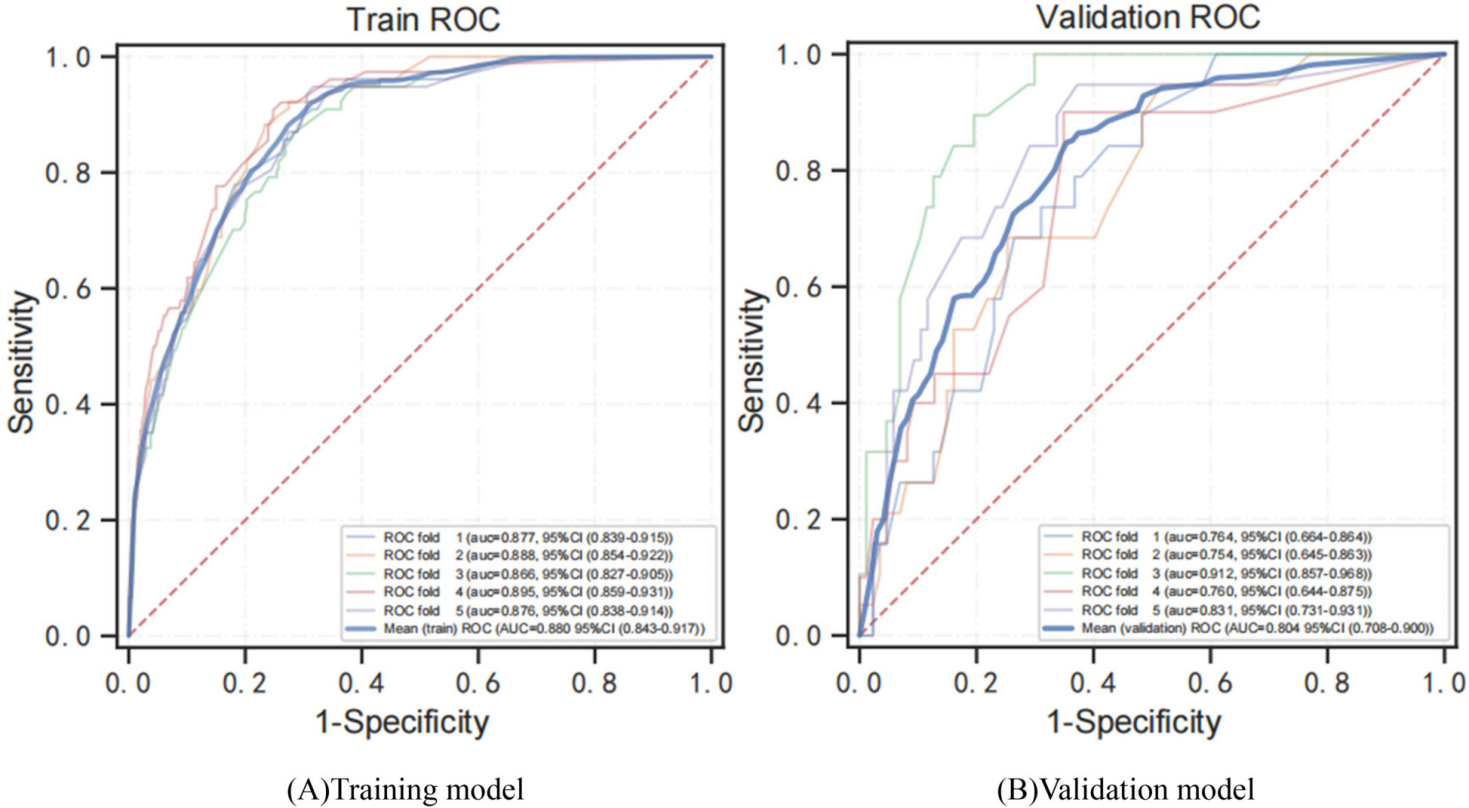
ROC curves for training and verification of the XGBoost model by 5-fold CV. **(A)** ROC analysis results of the XGBoost training model. **(B)** ROC analysis results of the XGB verification model. ROC curve, receiver operating characteristic curve; AUC, area under curve; XGBoost, adaptive boosting.

Figure 5 shows the evaluation of the XGBoost model. Based on a five-fold CV, the mean AUC for the XGBoost training and validation model was 0.880 (Figure 5A) and 0.804 (Figure 5B). The predicted probability of early cognitive impairment was positively correlated with the actual probability, and the XGBoost model had excellent calibration (*P* > 0.05).

### 3.5 DCA analysis of predictive model

**Figure 6:**
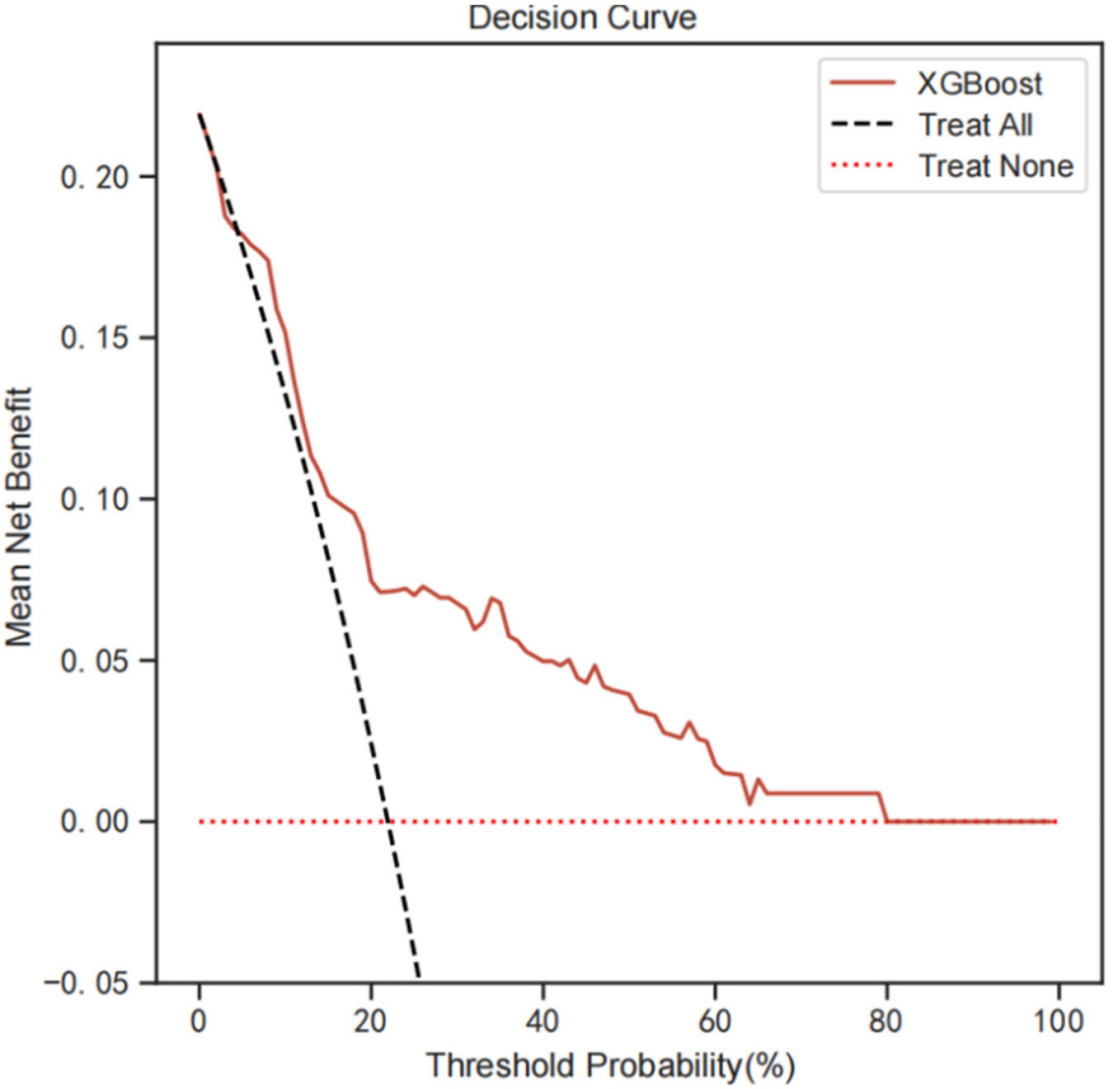
DCA analysis was performed to evaluate the clinical usefulness of the XGB model. The *y*-axis indicated the net benefit; the *x*-axis indicated the threshold probability. The solid red line shows the net benefit rate of the XGB forecast model. Within a certain threshold range, the XGB model has a higher net benefit. DCA, decision curve analysis.

To evaluate the clinical application of the model, we performed DCA analysis. Figure 6 show the threshold probability for XGBoost Model. Primary analysis demonstrated optimal performance within 15% (*Δ*net benefit = 0.041) - 25% (*Δ*net benefit = 0.068) threshold range. The maximum net benefit of 0.085 (95% CI: 0.062–0.109) was obtained at the 20% threshold. Specifically, at a threshold probability of 0.20 (representing 20% risk of early cognitive impairment), the XGBoost model demonstrated superior clinical utility with a maximum net benefit of 0.085 (95% CI: 0.062–0.109). This corresponds to identifying 8.5 true positive cases per 100 patients while avoiding unnecessary interventions in 14.7% of cases (false positives), translating to a risk-benefit ratio of 1:1.72.

### 3.6 Feature importance of predictive model

**Figure 7:**
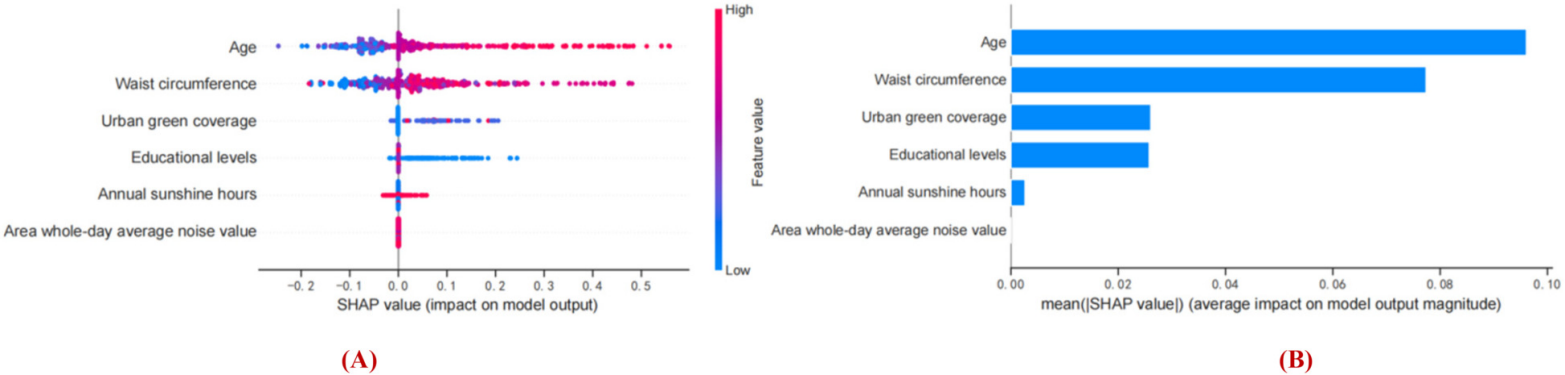
Interpretation of the obtained model based on SHAP analysis. **(A)** Prediction direction of model features. **(B)** Ranking the importance of model features. The vertical axis of the graph represents features and the horizontal axis represents SHAP observations. Each point represents a feature and a Shapley value, which represents the contribution of each feature to the model output. Feature values are represented by color, and feature importance is arranged from top to bottom along the *Y*-axis. Pink shows a positive association with early cognitive impairment in hypertension, blue shows a negative association with early cognitive impairment in hypertension.

Figures 7A, B show the feature importance of the XGBoost model based on SHAP analysis. SHAP values represent the combination of feature importance and feature effects. Figure 7B shows the order of importance of the features: age > waist circumference > urban green coverage > educational levels > hours of annual sunshine > area whole-day average noise. Current results indicate that age is the most important feature in predicting Shapley value. Aging is positively associated with Shapley value, and older age was more likely to be predicted to have cognitive impairment. Secondary to age is waist circumference. Having a larger waist circumference, higher area whole-day average noise (colored pink) was associated with Shapley values and was a positive predictor of early cognitive impairment in hypertension. Having lower urban green coverage and educational attainment, fewer hours of annual sunshine (blue color) were associated with Shapley values and were negative predictors of early cognitive impairment in hypertension. In summary, age, waist circumference, and area whole-day average noise were positive predictors of early cognitive impairment in hypertension, while urban green coverage, educational levels, and annual sunshine hours were negative predictors of cognitive impairment in hypertension. It suggests the threat of personal factors (older age, larger waist circumference, lower education level) and environmental factors (lower urban green coverage, fewer hours of annual sunshine, and higher area whole-day average noise value) to the risk of early cognitive impairment in hypertension.

## 4 Discussion

Our findings suggested that age, waist circumference, urban green coverage, educational levels, annual sunshine hours, and area whole-day average noise were significant predictors of early cognitive impairment in hypertension. Five ML models of XGBoost, LR, AdaBoost, GNB and SVM based on multiple predictors were developed and validated. The XGBoost model performed significantly better than other classifier models, with AUC (0.893), sensitivity (0.780), specificity (0.853), accuracy (0.837), and F1 score (0.627). DCA analysis shows that the current predictive model has excellent clinical practicability. These findings may provide evidence for diagnosis and risk prediction of early cognitive impairment in hypertension.

Evidence for the use of predictors in clinical practice remains highly controversial and evidence-based data is lacking to recommend a diagnostic assessment of an individual risk for preclinical stages of cognitive impairment. The predictive model of cognitive impairment based on risk factors is still in the exploratory stage, but promising. To date, most of the development of predictive models for cognitive impairment has focused on a single personal factor, while designs for environmental factors have not been reported. Recently, there has been growing recognition that cognitive decline is often attributed to a combination of risk factors (5), in which both personal and natural environmental factors are considered to be significant influencing factors on cognitive impairment. In this study, with the advantages of multicollinearity and overfitting among effective control variables (36), LASSO regression helped us screen for six predictors of early cognitive impairment in hypertension, including age, education level, waist circumference, annual sunshine duration, area whole-day average noise, and urban green coverage. Further SHAP analysis revealed the feature importance of the predictive model.

At present, exploring the relationship between personal factors and cognitive impairment is still mainstream. In this study, we observed that aging is the most important risk factor. Aging is a common risk factor for hypertension and cognitive decline. Multidimensional homeostasis dysfunction and impaired cellular stress recovery caused by aging can accelerate the destruction of the cerebral blood supply and blood-brain barrier in patients with chronic hypertension, leading to cognitive decline (3). Epidemiological evidence strongly support that hypertension is associated with cognitive decline, especially in midlife (37). A Finnish study reported that middle-aged people with SBP ≥ 160 mmHg were independently associated with a >2-fold increased risk of Alzheimer's disease (AD) (38). In older adults, however, the link between hypertension and cognitive function remains controversial (39). Waist circumference, an important measure of central obesity, was observed in this study as a secondary risk factor for cognitive impairment in hypertension. Several recent studies have also reported a strong correlation between waist circumference and cognitive decline (40–42). As waist circumference increases, chronic low inflammation associated with obesity can lead to multiple chronic diseases and neuroinflammation, which is an important pathway for cognitive decline (43). Previous studies have shown that that educational attainment can have a positive impact on cognitive function (44, 45). A similar conclusion was observed in our study, which showed an independent positive association between low educational attainment and cognitive impairment in hypertension. Emerging evidence suggests that the effect of education level on cognitive function in late-life is associated with promoting individual differences in cognitive ability that occur in early adulthood but persist into old age (44). Interestingly, we observed that patients with early cognitive impairment simultaneously exhibited larger waist circumference and a higher proportion of engaging in physical labor; however, this finding is not contradictory. Many of these individuals are manual laborers, often shift workers, who typically have higher alcohol consumption, larger waistlines, and increased energy intake (46). Evidence strongly links shift work to a higher risk of abdominal obesity (47), likely due to metabolic dysfunction from circadian rhythm disruption. Thus, these findings indicate shared pathophysiological mechanisms, such as metabolic disturbances from circadian dysrhythms, rather than contradictory outcomes.

Recently, increasing attention has been paid to the impact of environmental factors on cognitive function. In this study, we observed less annual sunlight hours as a risk factor for cognitive impairment in hypertension. Sunlight exposure is an important factor affecting circadian rhythm and sleep-wake time (48), and disruption of circadian rhythm and lack of sunlight are related to mood fluctuations (49), all of which may have an impact on cognitive function. Both Zhu et al. (50) and Chantranupong et al. (51) have reported that sunlight can alter mood, behavior, and cognition, while enhancing learning and memory. Moreover, previous studies consistently indicated that long-term residential natural sunlight exposure is associated with a lower risk of cognitive decline (49, 52–54). In general, less annual sunshine means that patients live in rainy and hazy weather, high humidity, low oxygen content in the air, low sympathetic excitability, and easy to produce mood swings. In addition, the relative decline in outdoor activities is not conducive to interpersonal interaction, and may induce insomnia, anxiety, and depression, which can lead to cognitive decline. Area whole-day average noise is another important environmental factor in current models for predicting cognitive impairment in hypertension. Similar to our study, a large number of scholars have also demonstrated that chronic noise exposure (CNE) is an independent risk factor for cognitive impairment (55–58). Animal research has shown that CNE can cause neuroendocrine disorders, overactivation of sympathetic regions of the autonomic nervous system, and an increase in stress hormones that affect the brain and behavior (59), meanwhile, noise exposure can also trigger endothelial and neuronal dysfunction, which activates inflammatory and oxidative stress pathways (60), which may explain the adverse cognitive effects of noise. In addition, anxiety and depression caused by environmental noise exposure have also become longitudinal predictors of impaired cognitive function (61, 62). Green spaces are conducive to better cognitive function. In this study, urban green coverage was also identified as a significant factor in early cognitive impairment in hypertension. Urban greening benefits residents by regulating climate, air quality and water resources, and a comfortable experience can bring a good mood to residents. Hu et al. (63) reported that participants with the most green space had a 20% lower risk of cognitive impairment [hazard ratio (HR): 0.80, 95% CI: 0.73–0.89]. Recent systematic review and meta-analysis (57) suggested that the protective effect of more greenness (OR = 0.97, 95% CI: 0.95–0.995) on cognitive function. In addition, a population-based cohort study (9) also highlighted the protective effects of greenness on cognitive function. Taken together, our findings may shed light on future research to better understand the role of natural environmental factors in the association between cognitive impairment.

Today, ML has provided a new paradigm for monitoring cognitive impairment, but there is little evidence of its application to hypertensive cognitive impairment. Hu et al. (21) established a logistic regression model for Chinese community-dwelling elderly with normal cognition using age, instrumental activities of daily living, marital status, and baseline cognitive function, with a consistency index of 0.814 (95% CI 0.781–0.846). Yadgir et al. (22) developed an ML model to assist in screening for cognitive impairment in the emergency department, the best performing algorithm was the XGBoost model with an AUC of 0.72, sensitivity of 0.73, and specificity of 0.64. Tan et al. (34) developed an ensemble model based on three classifiers using age, race, highest education, and neuroimaging markers with an AUC of 0.80, accuracy of 0.83, F1 score of 0.87, sensitivity of 0.86, and specificity of 0.74. Liu et al. (24) noted that random forest models had high accuracy for all outcomes at Year 2 (AUC = 0.81), Year 4 (AUC = 0.79), and cross-sectional Year 4 (AUC = 0.80). Ciarmiello et al. (64) developed a deep learning model using radiomic and amyloid PET load, with an AUC of 0.71, accuracy of 0.57, and F1 score of 0.48. Na et al. (65) developed a gradient enhancer (GBM) model to predict cognitive impairment after 2 years, which showed good performance with sensitivity of 0.967, specificity of 0.825, and AUC of 0.921. Similar ML predictive models for cognitive impairment have also been reported in other studies as well (66–69). According to the latest systematic review (70), SVM is the most common machine model developed to predict dementia, with an average accuracy of 75.4%, while convolutional neural network (CNN) has a higher average accuracy of 78.5%. In this study, XGBoost showed the best predictive performance in our prediction models based on five classifiers, with AUC (0.893), sensitivity (0.780), specificity (0.853), accuracy (0.837), and F1 score (0.627). The several recent studies conducted by Du et al. (71) have consistently demonstrated the superior performance of the XGBoost model in disease prediction and diagnosis. Our developed model has some advantages. Our previous study revealed that hip circumference, age, education level, and physical activity are significant predictors of early cognitive impairment in individuals with hypertension (72). The XGB model, which is based on hip circumference, age, education level, and physical activity, demonstrated a stronger predictive effect on the risk of cognitive impairment in hypertensive patients. It achieved an AUC of 0.88, F1 score of 0.59, accuracy of 0.81, sensitivity of 0.84, and specificity of 0.80. However, it should be noted that environmental exposures were not included in this analysis. In this study, we investigated a multi-center population of hypertensive patients and integrated natural environmental factors into the model development process. Subsequently, we identified the most superior predictive model for further analysis.

Inevitably, the limitations of the current study must be considered. First, we only analyzed patient data from four hospitals in Shandong Province, with a limited sample size, and this data may not cover all regions and residents. Second, we selected only a few important individual and natural environmental factors as predictors for model development and may have overlooked some other important indicators, such as lifestyle (73), genetics (74), air pollution (75), and drinking water quality (76). Third, the design of the cross-sectional study makes it difficult to determine causality between hypertension and cognitive impairment. Fourth, although the MMSE questionnaire assessment has a better diagnostic value for early cognitive impairment, the Montreal Cognitive Assessment (MoCA) may be more appropriate for screening in this study (77). Finally, given the variability of natural environmental factors at different latitudes, the generalizability of current results to populations at other latitudes remains limited. Nevertheless, we conducted a comprehensive assessment of cognitive function in hypertensive patients from multiple regions, and used detailed residence information to link physical environment data to each patient. To our knowledge, this is the first reliable ML predictive model of early cognitive impairment in hypertension developed and validated based on simple personal and natural environmental factors, which indeed provides a new perspective for diagnostic decision-making and prevention of cognitive impairment in hypertension in a clinical setting. The preliminary findings warrant further validation through longitudinal cohort studies to establish temporal causality and robustness of observed patterns.

## 5 Conclusions

In conclusion, the XGBoost model based on age, waist circumference, urban green coverage, educational levels, annual sunshine hours, and area whole-day average noise has superior predictive performance and clinical practicability, which provides a reliable and economical tool for the diagnosis and risk prediction of early cognitive impairment in hypertension. Further investigations, including the design of large-sample multicenter research, multimodal and multidimensional data, predictor thresholds, and model optimization, should be considered in future research.

## Data Availability

The raw data supporting the conclusions of this article will be made available by the authors, without undue reservation.
